# RAS mutation leading to acquired resistance to dabrafenib and trametinib therapy in a multiple myeloma patient harboring BRAF mutation

**DOI:** 10.1002/jha2.8

**Published:** 2020-08-06

**Authors:** Baptiste Le Calvez, Yannick Le Bris, Guillaume Herbreteau, Bastien Jamet, Céline Bossard, Benoit Tessoulin, Thomas Gastinne, Béatrice Mahé, Viviane Dubruille, Nicolas Blin, Chloé Antier, Olivier Theisen, Françoise Kraeber‐Bodéré, Steven Le Gouill, Marie C. Béné, Philippe Moreau, Cyrille Touzeau

**Affiliations:** ^1^ Department of Hematology University Hospital Nantes France; ^2^ Hematology Biology University Hospital Nantes France; ^3^ Biochemistry Laboratory University Hospital Nantes France; ^4^ Department of Nuclear Medicine University Hospital Nantes France; ^5^ Service d'Anatomie et Cytologie Pathologiques, INSERM, CRCINA Université de Nantes, CHU Nantes Nantes F44000 France; ^6^ CRCINA, INSERM, CNRS Université d'Angers Université de Nantes Nantes France; ^7^ Site de Recherche Intégrée sur le Cancer (SIRIC) « ILIAD » INCA‐DGOS‐Inserm_12558 Nantes France

**Keywords:** BRAF, dabrafenib, multiple myeloma, RAS, trametinib

## Abstract

Multiple myeloma (MM) is still considered incurable and new therapeutic approaches are therefore needed. Deep‐sequencing analysis revealed the presence of *BRAF* mutations in up to 15% of patients. The clinical experience of *BRAF*‐targeted therapy in myeloma patients harboring *BRAF* mutation is still limited. We here report the case of a patient with penta‐refractory (bortezomib, lenalidomide, carfilzomib, pomalidomide, and daratumumab) MM with extramedullary BRAF‐mutated disease that achieved clinical response to dual BRAF and MEK inhibition. At the time of disease progression, gene sequencing analysis of the tumor at the time of progression demonstrated a clonal evolution with emergence of a *NRAS* mutation and persistence of *BRAF* and *TP53* mutations. Backtracking of the *NRAS* mutation was performed by digital polymerase chain reaction on the baseline biopsy and identified the pre‐existence of the *NRAS* at a subclonal level. This observation is the first report of acquired NRAS mutation leading to resistance to dual BRAF/MEK inhibitors in MM. These data suggest that a systematic search for RAS mutations using highly sensitive techniques should be performed before considering targeted therapy in relapsed myeloma with BRAF mutation.

Multiple myeloma (MM) is still considered incurable and the outcome of patients with triple‐class refractory (proteasome inhibitors, immunomodulatory drugs, and anti CD38 monoclonal antibodies) disease remains very poor [1]. New therapeutic approaches with distinct mechanism of action are therefore needed. Deep‐sequencing analysis revealed the presence of *BRAF* mutations in 11‐15% of patients [2, 3, 4, 5]. The clinical experience of BRAF‐targeted therapy in myeloma patients harboring *B‐RAF* mutation is still limited [6, 7, 8, 9, 10]. Here, we report the case of a patient with triple‐class refractory myeloma harboring *BRAF* mutation that achieved clinical response to dual BRAF and MEK inhibition but experienced early disease progression related to a clonal evolution involving RAS.

A 60‐year‐old male was diagnosed with symptomatic free‐light chain kappa MM in November 2018. At the time of diagnosis, he presented with anemia, bone lesions, hypercalcemia, and circulating plasma cells (5%). Revised International Scoring System score was 3. The Fluorescence in situ Hybridization analysis identified a 1p32 deletion. Front‐line therapy consisted on the triplet bortezomib‐lenalidomide‐dexamethasone (VRd). After three cycles, the patient presented disease progression with new bone lesions and appearance of extramedullary disease involving skin (Figures 1A and 1B). At that time, the triplet combination carfilzomib‐pomalidomide‐dexamethasone (KPd) was initiated. After two cycles, disease progression was confirmed and the patient started daratumumab‐cyclophosphamide and dexamethasone. Again, therapy was ineffective with extramedullary disease progression. A skin biopsy confirmed the presence of clonal plasma cell involvement expressing BRAF V600E mutant (Figure 1C). Molecular analysis revealed the presence of a *BRAF* mutation (p.Val600Glu; c.1799T>A, 43%) together with a *TP53* mutation (p.Cys176Gly; c.526T>G; 91%). In this context of penta‐refractory (bortezomib, lenalidomide, carfilzomib, pomalidomide, and daratumumab) MM with extramedullary BRAF‐mutated disease, the combination of dabrafenib (an oral BRAF inhibitor) associated with trametinib (an oral MEK inhibitor) was initiated. The patient presented a rapid clinical response with complete disappearance of skin lesions. No drug‐related adverse event has been reported. Two months after treatment initiation, the patient experienced disease progression with recurrence of extramedullary disease. A new skin biopsy at the same site confirmed clonal plasma cells recurrence. Gene sequencing revealed the emergence of a mutation of *NRAS* p.Gly12Asp (c.35G>A, 47%) and the persistence of *TP53* (90%) and *BRAF* (48%) mutations already present before treatment with the combination of dabrafenib plus trametinib. Backtracking of the *NRAS* mutation was performed by digital polymerase chain reaction on the material sampled before treatment initiation and identified the pre‐existence of the *NRAS* p.G12D mutation at a subclonal level, with a variant allele frequency of 0.1% (Figure 2). At time of disease progression on dabrafenib plus trametinib, the patient was treated with panobinostat in addition with bortezomib and dexamethasone. No clinical response was obtained and the patient died a few weeks later due to disease progression.

**FIGURE 1:**
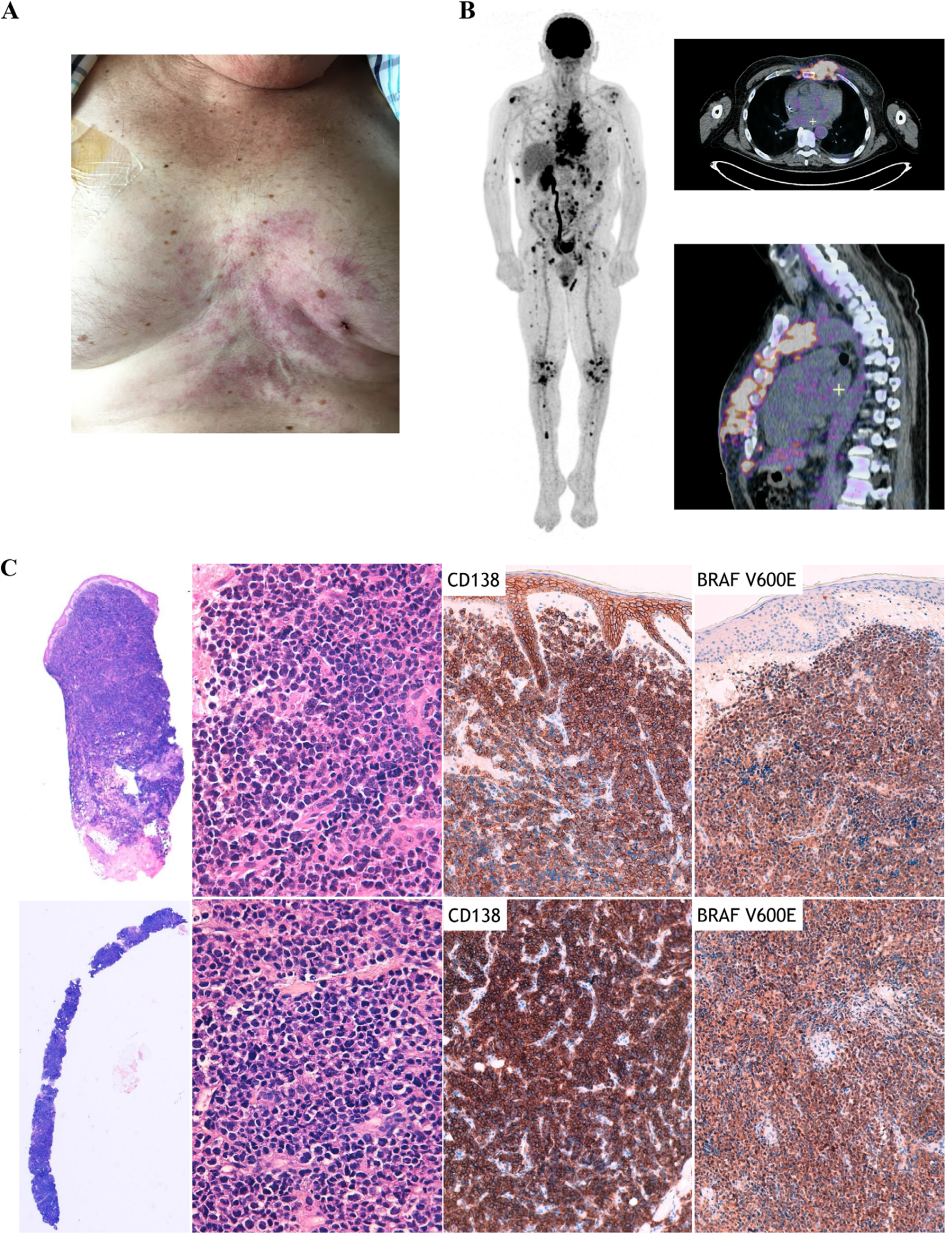
Patient disease with extramedullary involvement. A, Patient with relapsed refractory multiple myeloma with extramedullary skin involvement. B, ^18^FDG PET‐CT imaging showing extramedullary involvement and multiple focal lesions on axial and appendicular skeleton. C, Morphology and immunochemistry of skin biopsies performed before and at the time of disease progression under BRAF+MEK inhibition. Upper part: pretreatment skin biopsy showing a diffuse cutaneous and subcutaneous infiltration by large tumor cells (a, ×1.25 magnification; b, ×40 magnification). Immunohistochemistry demonstrated strong expression of CD138 and BRAF V600E by tumor cells (c, ×200 magnification; d, ×200 magnification). Lower part: second biopsy with the same morphological and immunophenotypic characteristics (e, ×1.25 magnification; f, ×40 magnification; g, ×200 magnification; h, ×200 magnification)

**FIGURE 2:**
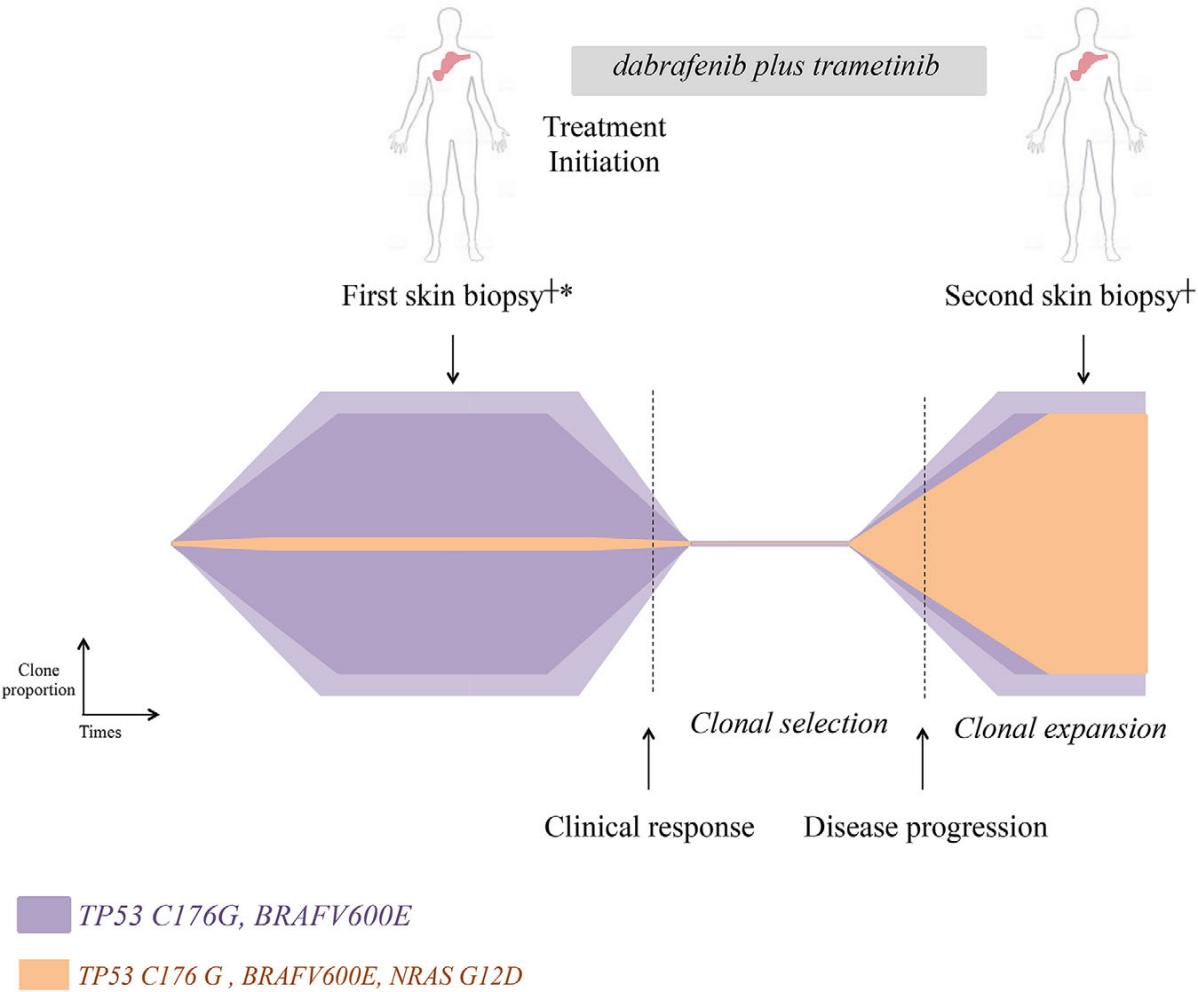
Schematic representation of RAS/RAF clonal evolution. Methods: ^┼^DNA was extracted from fresh skin biopsies with a Maxwell RSC DNA FFPE Kit (Promega, Madison, WI, USA) and sequenced with QIAseq Targeted DNA Custom Panel (Qiagen, Hilden, DE, USA).*Backtracking of the *NRAS* p.G12D mutation was performed using high‐sensitivity digital polymerase chain reaction (QuantStudio 3D, Thermo Fisher Scientific, Waltham, MA, USA)

To date, the clinical response to BRAF‐targeted therapy in relapsed MM patients harboring *BRAF* mutation has been reported in only a few patients [8, 9, 10, 11]. The efficacy of the BRAF inhibitor vemurafenib as single agent has been reported in four patients with relapsed extramedullary MM with *BRAF* p.V600E mutation that failed both proteasome inhibitor and immunomodulatory agents [6, 9, 10]. In this context, vemurafenib provided partial responses during from 2 to 8 months. In melanoma, dual rather than single inhibition of the MAPK pathway (BRAF and MEK co‐inhibition) has been shown to significantly prolong the duration of response to therapy [11]. In myeloma, the combination of BRAF and MEK co‐inhibition (vemurafenib and cobimetinib, dabrafenib and trametinib) has been reported in four patients with advanced MM and *BRAF* p.V600E mutation and resulted in transient partial response [7, 8]. Here, we obtained a transient clinical response to the combination of dabrafenib and trametinib in a penta‐refractory patient with extramedullary disease harboring both a *BRAF* p.V600E mutation in addition with *TP53* mutation.

Molecular mechanisms leading to resistance to BRAF inhibition in myeloma still need to be elucidated. Recently, the case of a clonal evolution involving mutation within the capicua transcriptional repressor (*CIC*) gene leading to BRAF and MEK inhibitors resistance has been described in a MM patient [7]. In melanoma, mutations of genes of the *RAS*‐family have been shown to be a major mechanism of resistance to *BRAF* inhibition [12]. A similar mechanism of resistance has been described in a MM patient harboring BRAF mutation treated with vemurafenib [9]. Interestingly, the authors documented the acquisition of distinct NRAS mutations in three different extramedullary lesions at time of treatment failure. In the present case, gene sequencing analysis of the tumor at the time of progression demonstrated a clonal evolution with emergence of a *NRAS* mutation and persistence of *BRAF* and *TP53* mutations. In the present case, a hypothesis about the early progression is that the therapeutic pressure exerted by the combination of dabrafenib plus trametinib could have resulted in the selection of a pre‐existing resistant subclone harboring both *BRAF* and *NRAS* mutations. Indeed, in *RAS*‐mutated cells, BRAF inhibitors are known to induce paradoxical activation of the MAPK pathway via the formation of a BRAF/CRAF heterodimer [13]. This effect can be limited by the combination with a MEK inhibitor [14], nevertheless tumor escape after 2 months of treatment with this combination suggests that in MM cells carrying an activating mutation of a *RAS* gene, MEK inhibition is insufficient to block the excessive oncogenic activation of the MAPK pathway due to CRAF trans‐activation. Although the first description in melanoma of a mutual exclusivity of *NRAS* and *BRAF* mutations, more recent studies have shown that their co‐occurrence was possible in the same tumor before treatment [14]. However, if these mutations can coexist in the same melanoma lesion, data suggest that these mutations may be present in separate cell subclones, and do not coexist within the same cell. This characteristic does not seem to apply to MM, where the co‐occurrence of MAPK pathway mutations has been previously described [15]. Here, it appears that they coexisted within the same cell clone before treatment.

This observation confirms the implication of NRAS mutation in the resistance to BRAF/MEK targeted therapy in MM. A Phase 1 clinical trial is currently evaluating the combination of dabrafenib and trametinib in relapsed MM patients with BRAF mutation (NCT03091257). The ongoing BIRMA trial (NCT02834364) is evaluating encorafenib (BRAF inhibitor) in combination with binimetinib (MEK inhibitor) in BRAFV600E‐mutated MM. The present case suggests performing a systematic search for RAS mutations using highly sensitive techniques in order to identify potential biomarker of resistance to those targeted therapies. The present case report highlights the need of innovative strategies to overcome resistance to BRAF/MEK co‐inhibition in multiple myeloma.

## CONFLICT OF INTEREST

The authors declare no conflict of interest.

## AUTHOR CONTRIBUTIONS

BLC, YLB, PM, and CT wrote the manuscript. YLB, GH, CB, and OT performed experiments. CT, SLG, PM, BM, TG, NB, and JSB treated the patient. BJ provided imaging data. All the authors critically reviewed the manuscript.
